# Clinical significance of D-dimer levels during acute period in ischemic stroke

**DOI:** 10.1186/s12959-023-00496-1

**Published:** 2023-05-09

**Authors:** Ki-Woong Nam, Hyung-Min Kwon, Yong-Seok Lee

**Affiliations:** 1 Department of Neurology, Seoul Metropolitan Government-Seoul National University Boramae Medical Center, Seoul, South Korea; 2 Department of Neurology, Seoul National University College of Medicine, Seoul, Korea

**Keywords:** D-dimer, Ischemic strok, Coagulopathy, Early neurological deterioration, Prognosis

## Abstract

**Background:**

Initial D-dimer level is a well-known prognostic parameter in patients with acute ischemic stroke (AIS). However, there have been no studies on the clinical significance of follow-up D-dimer levels. In this study, we evaluated the association between initial and follow-up D-dimer levels and early neurological deterioration (END) in patients with AIS.

**Methods:**

We included consecutive patients with AIS who had a positive initial D-dimer test (> 0.55 mg/L) between March 2021 and November 2022. The follow-up D-dimer test was performed on the 7th day after hospitalization and on the day of discharge if discharged earlier. END was defined as an increase of ≥ 2 in the total NIHSS score, or ≥ 1 in the motor NIHSS score within the first 7 days of admission. As medical conditions closely associated with the initial and follow-up D-dimer levels in AIS patients, we also evaluated the history of cancer, active cancer, and venous thromboembolism (VTE) that occurred during hospitalization together.

**Results:**

A total of 246 patients with AIS were evaluated (median age: 87 years, male: 56.5%). In multivariable logistic regression analysis, the initial D-dimer level was closely associated with END after adjusting for confounders (adjusted odds ratio [aOR]: 1.48, 95% CI: 1.06–2.05). The follow-up D-dimer level also showed a close correlation with END (aOR: 1.60, 95% CI: 1.16–2.20). Regarding the analysis of the association between D-dimer levels and underlying cancer or VTE, the initial D-dimer level showed a statistically significant positive relationship only with active cancer (*P* = 0.024). On the other hand, the follow-up D-dimer level was found to be statistically significantly associated with a history of cancer (*P* = 0.024), active cancer (*P* = 0.001), and VTE (*P* = 0.001).

**Conclusions:**

Initial and follow-up D-dimer levels were associated with END in AIS patients. Particularly, the follow-up D-dimer level showed a clear correlation not only with END but also with the underlying cancer or the occurrence of VTE during the acute period.

**Supplementary Information:**

The online version contains supplementary material available at 10.1186/s12959-023-00496-1.

## Background

D-dimer is a by-product of the degradation of cross-linked fibrin by plasmin [1, 2]. It is mainly produced by secondary fibrinolysis after thrombus formation and elevated D-dimer levels reflect ongoing or potential thrombus formation:[1–4] therefore, D-dimer has been used in the clinical diagnosis of various thromboembolic diseases [5, 6]. The most representative diseases are deep vein thrombosis or pulmonary embolism, and D-dimer is widely used in conjunction with clinical scales to exclude these diseases in patients with low clinical probability [7, 8]. D-dimer has been also proposed as a diagnostic tool for various cardiovascular, cerebrovascular, and aortic diseases, but has not gained as much consensus as venous thromboembolism yet [9–11].

D-dimer has also been used as an indicator in patients with ischemic stroke [2]. Its level is closely related to the size or severity of stroke lesions and short-term and long-term prognosis [12–17]. Additionally, the D-dimer level indicates the etiological mechanism of stroke [3, 18, 19]. D-dimer levels are higher in cardiogenic strokes that form fibrin-rich clots than in strokes caused by other mechanisms 18–21]. In strokes caused by large artery disease that form a platelet-rich thrombus or small vessel occlusion based on lipohyalinosis, D-dimer elevation was relatively insignificant [4, 20, 21]. Further, hidden malignancy has recently been suggested as one of the major etiologies of cryptogenic stroke, especially Embolic Stroke of Undetermined Source (ESUS), and its frequency can go up to 6.5% 22]. In these patients, D-dimer was found to be the most potent diagnostic marker and plays an important role as a predictor of early neurological deterioration (END), recurrence, and mortality [21, 23, 24].

Most studies on D-dimer levels in patients with ischemic stroke focus only on the initial level. However, similar to an acute phase reactant, D-dimer has a natural course of increasing immediately after an acute ischemic stroke (AIS) and then gradually decreasing thereafter [1, 5, 13, 14]. In addition, there are reports that the level of change of this increase and decrease is different depending on the stroke mechanism [5, 21]. Complications occurring during the acute phase, comorbidity, treatment, and response to treatment may also affect the follow-up D-dimer level 21]. Therefore, it is thought that changes in D-dimer levels during the acute period will have a close relationship with the early outcomes of patients with AIS, but more research is still needed.

In this study, we evaluated the association between initial D-dimer levels and END in AIS patients with elevated (positive) results on initial tests. Furthermore, using the follow-up D-dimer measurement (within 7 days), we identified the clinical significance of the changes in D-dimer levels. To determine the mechanism by which initial and follow-up D-dimer levels affect the prognosis of patients with AIS, we also analyzed D-dimer levels according to stroke severity, mechanisms, and complications.

## Methods

### Study population

As part of a consecutive stroke registry at a large stroke center in Korea (Seoul Metropolitan Government-Seoul National University Boramae Medical Center [SMG-SNUBMC]), we included consecutive patients with AIS who had a positive initial D-dimer test (> 0.55 mg/L) between March 2021 and November 2022 (n = 308). Since 2021, SMG-SNUBMC has been conducting a clinical protocol to measure follow-up D-dimer levels after 7 days in all AIS patients whose initial D-dimer levels exceeded the normal range (i.e., positive result). In case of discharge within 7 days, D-dimer level was measured at the time of discharge. Patients who met the following criteria were excluded: (1) age < 20 years (n = 5), (2) arrival more than 24 h after symptoms onset (n = 25), and (3) received therapeutic thrombolytic therapy (n = 32). Altogether, 246 patients were included in the final analysis.

This retrospective cross-sectional study was approved by the Institutional Review Board (IRB) of SMG-SNUBMC (IRB number: 06-2021-0064), which waived the requirement for written informed consent because of the retrospective design and use of de-identified information. All experiments were performed in accordance with the Declaration of Helsinki and all relevant guidelines and regulations. All data and materials related to this article are included in the main text and supplementary materials.

### Demographic, clinical, and laboratory assessment

We evaluated baseline demographic and clinical factors, including age, sex, onset to visit time, hypertension, diabetes, dyslipidemia, ischemic heart disease, atrial fibrillation, current smoking, history of stroke, initial severity and mechanism of stroke, and use of anticoagulants [25]. The initial stroke severity was rated using the National Institutes of Health Stroke Scale (NIHSS) score on a daily basis from admission to discharge by neurologists who were not involved in this study [23]. The NIHSS score is a scale system used to assess the severity of hemorrhagic or ischemic stroke. To do so, 11 items are evaluated and assigned a score ranging from 0 to 42 (Additional file 1: Table S1)[26]. The mechanisms of stroke were divided according to the Trial of Org 10,172 in Acute Stroke Treatment (TOAST) classification (Additional file 2: Table S2) 27]. As a main outcome variable, END was defined as an increase of ≥ 2 in the total NIHSS score, or ≥ 1 in the motor NIHSS score within the first 7 days of admission [23].

There are various medical conditions that can cause an increase in D-dimer levels [28]. Among them, we included the following medical conditions that could significantly impact the prognosis and initial and follow-up D-dimer levels during the acute phase of ischemic stroke as variables: history of cancer, active cancer, and venous thromboembolism (VTE). Active cancer is recently emerging as one of the major underlying mechanisms of ischemic stroke, and it is known to be associated with a significant increase in D-dimer levels and the worst prognosis of stroke. As defined in previous studies, we defined active cancer as a new diagnosis, recurrence, or progression of cancer, or treatment for cancer within 6 months before enrollment [23, 24] Due to the immobility caused by stroke, VTE is one of the most common complications experienced by AIS patients and is also a condition that causes a significant increase in initial and follow-up D-dimer levels. In this study, VTE was defined as deep vein thrombosis and pulmonary embolism confirmed by tests during hospitalization.

Laboratory examinations were also conducted within the first 24 h of admission, including glucose profile, lipid profile, white blood cell (WBC) count, high-sensitivity C-reactive protein (hs-CRP), and D-dimer levels. D-dimer levels were measured using an immunological assay (Sysmex® CS-5100, Siemens Healthcare GmbH, Erlangen, Germany). In our institution, the upper limit of the normal range for D-dimer test was 0.55 mg/L. Therefore, all the participants in this study had an initial D-dimer exceeding 0.55 mg/L, and a follow-up D-dimer test was performed on the 7th day of admission.

### Statistical analysis

All statistical analyses were performed using SPSS version 23.0 (IBM Corp. Chicago, IL, USA). Continuous variables with normal distributions are shown as mean ± standard deviation, and the others are presented as median + interquartile range. Continuous variables with skewed data were transformed to log scales. To determine the characteristics associated with initial and follow-up D-dimer levels, we examined the association between D-dimer levels and stroke severity, mechanism, underlying cancer, VTE, and anticoagulant use. Simple linear regression analysis was used for this analysis.

Univariate analyses were conducted to identify the possible predictors of END. We used Student’s *t*-test or the Mann-Whitney *U*-test for continuous variables and the chi-squared test for categorical variables. Variables with *P* < 0.10 from the results of univariate analyses were introduced as confounders in the multivariable logistic regression analysis. Since D-dimer level is known to have a close relationship with stroke severity,[29] we also checked whether the initial stroke severity has a biological interaction with the effect of D-dimer on END. To do this, new variables of [initial NIHSS score x initial D-dimer] and [initial NIHSS score x follow-up D-dimer] were created as interaction terms and introduced together into multivariable analyses.

Finally, we checked whether the initial and follow-up D-dimer levels had a quantitative relationship with END and comorbidities/complications that could cause thromboembolic events. The initial and follow-up D-dimer levels were divided into three groups based on tertile values, and END, history of cancer, active cancer, and VTE frequencies between groups were compared using chi-squared test and linear-by-linear association method. All variables with *P* < 0.05 were considered significant.

## Results

Altogether 246 AIS patients were evaluated (median age: 78 years; male: 56.5%). END occurred in 58 (23.6%) patients, and the median initial NIHSS score was 4 [1–8]. The median value of the initial D-dimer was 1.25 [0.80–2.19] mg/L, and the median value of the follow-up D-dimer after 7 days was 1.30 [0.76–2.61] mg/L. Among our study population, 52 (23.2%) patients were discharged before 7 days and follow-up D-dimer was measured on the day of discharge. Other detailed baseline characteristics are presented in Table 1.

**Table 1:** Baseline characteristics of the study population (n = 246)

Demographic & clinical factors	
Age, y [IQR]	78 [68–83]
Sex, male, n (%)	139 (56.5)
Visit time, h	4.0 [1.5–12.0]
Hypertension, n (%)	164 (66.7)
Diabetes, n (%)	78 (31.7)
Dyslipidemia, n (%)	101 (41.1)
Ischemic heart disease, n (%)	39 (15.9)
Atrial fibrillation	61 (24.8)
Current smoking, n (%)	47 (19.1)
History of stroke, n (%)	65 (26.4)
Stroke mechanism, (%)	
Large artery atherosclerosis	74 (30.1)
Small-vessel occlusion	39 (15.9)
Cardioembolism	67 (27.2)
Other determined	10 (4.1)
Undetermined	48 (19.5)
Two or more	8 (3.3)
Initial NIHSS score [IQR]	4 [1–8]
History of cancer, n (%)	35 (14.2)
Active cancer, n (%)	21 (8.5)
Venous thromboembolism, n (%)^*^	18 (28.6)
Use of anticoagulants, n (%)	48 (19.5)
**Laboratory factors**	
HbA1c, % [IQR]	5.9 [5.6–6.5]
Fasting glucose, mg/dL [IQR]	99 [88–117]
Total cholesterol, mg/dL [IQR]	162 [135–190]
White blood cell, x 10^3^/uL [IQR]	7.59 [5.86–9.67]
High-sensitivity CRP, mg/dL [IQR]	0.21 [0.07–0.85]
Initial D-dimer, mg/L [IQR]	1.25 [0.80–2.19]
Follow-up D-dimer, mg/L [IQR]	1.30 [0.76–2.61]
Change in D-dimer, mg/L [IQR]	-0.03 [-0.43 -0.51]
**Outcome factors**	
Early neurological deterioration, n (%)	58 (23.6)

NIHSS = National Institutes of Health Stroke Scale, CRP = C-reactive protein

^*^This variable was measured in 63 participants who underwent the evaluation

Both initial and follow-up D-dimer levels were positively correlated with initial NIHSS score, END, other determined stroke, cancer, active cancer, and VTE. In addition, the initial D-dimer level showed a positive correlation with cardioembolic stroke and a negative correlation with small-vessel occlusion stroke. The follow-up D-dimer level was negatively correlated with large artery atherosclerotic stroke (Table 2).

**Table 2:** Correlation between initial/follow-up D-dimer levels and stroke severity, mechanism, and complications during the acute phase: simple linear regression analysis

	Initial D-dimer level (mg/L)^*^		Follow-up D-dimer level (mg/L)^*^	
	*β* (95% CI)	*P* value	*β* (95% CI)	*P* value
Initial NIHSS score [IQR]	0.036 (0.020 to 0.052)	< 0.001	0.032 (0.015 to 0.049)	< 0.001
Early neurological deterioration, (%)	0.470 (0.197 to 0.743)	0.001	0.604 (0.321 to 0.888)	< 0.001
Stroke mechanism, (%)				
Large artery atherosclerosis	-0.214 (-0.473 to 0.044)	0.104	-0.308 (-0.578 to -0.038)	0.026
Small-vessel occlusion	-0.408 (-0.729 to -0.087)	0.013	-0.274 (-0.613 to 0.065)	0.113
Cardioembolism	0.291 (0.025 to 0.558)	0.032	0.131 (-0.151 to 0.413)	0.361
Other determined	1.344 (0.767 to 1.921)	< 0.001	1.434 (0.830 to 2.039)	< 0.001
Undetermined	0.019 (-0.281 to 0.318)	0.903	0.154 (-0.160 to 0.467)	0.335
Cancer, n (%)	0.584 (0.252 to 0.915)	0.001	0.575 (0.226 to 0.924)	0.001
Active cancer, n (%)	0.830 (0.418 to 1.242)	< 0.001	1.017 (0.590 to 1.443)	< 0.001
Venous thromboembolism, n (%)^†^	0.759 (0.177 to 1.341)	0.011	1.128 (0.606 to 1.650)	< 0.001
Use of anticoagulants, n (%)	0.291 (-0.006 to 0.588)	0.055	0.014 (-0.300 to 0.328)	0.931

NIHSS = National Institutes of Health Stroke Scale

^*^These variables were transformed into a log scale

^†^This variable was measured in 63 participants who underwent the evaluation

In univariate analysis, END was significantly associated with other determined strokes, initial NIHSS score, fasting glucose level, white blood cell (WBC) counts, and initial and follow-up D-dimer levels (Table 3). Multivariable logistic regression analysis showed that the initial D-dimer level was closely associated with END after adjusting for confounders (adjusted odds ratio [aOR] = 1.48, 95% confidence interval [CI]: 1.06–2.05, *P* = 0.020). The follow-up D-dimer levels also showed a close correlation with END (aOR = 1.60, 95% CI: 1.16–2.20, *P* = 0.004; Table 4). When analyzing the biological interaction with the initial NIHSS score, the effect of the initial D-dimer level on END showed a statistically significant interaction with the initial NIHSS score (*P* = 0.047). In contrast, follow-up D-dimer levels seemed to act on END regardless of the initial NIHSS score (*P* = 0.256; Additional file 3: Table S3).

**Table 3:** Comparisons of baseline characteristics of patients with and without early neurological deterioration

	No END (n = 188)	END (n = 58)	*P* value
Age, y [IQR]	77 [68–82]	79 [67–85]	0.496
Sex, male, n (%)	111 (59.0)	28 (48.3)	0.148
Visit time, d [IQR]	4.0 [1.5–13.0]	3.8 [1.5–10.0]	0.718
Hypertension, n (%)	123 (65.4)	41 (70.7)	0.457
Diabetes, n (%)	58 (30.9)	20 (34.5)	0.603
Dyslipidemia, n (%)	83 (44.1)	18 (31.0)	0.076
Ischemic heart disease, n (%)	28 (14.9)	11 (19.0)	0.458
Atrial fibrillation, n (%)	43 (22.9)	18 (31.0)	0.208
Current smoking, n (%)	40 (21.3)	7 (12.1)	0.119
History of stroke, (%)	50 (26.6)	15 (25.9)	0.912
Stroke mechanism, (%)			0.024
Large artery atherosclerosis	54 (28.7)	20 (34.5)	0.403
Small vessel occlusion	34 (18.1)	5 (8.6)	0.084
Cardioembolism	49 (26.1)	18 (31.0)	0.457
Other determined	4 (2.1)	6 (10.3)	0.006
Undetermined	41 (21.8)	7 (12.1)	0.102
Two or more	6 (3.2)	2 (3.4)	0.923
Initial NIHSS score [IQR]	3 [1–7]	7 [3–13]	< 0.001
Use of anticoagulants, n (%)	35 (18.6)	13 (22.4)	0.524
Hemoglobin A1c, % [IQR]	5.9 [5.6–6.5]	5.9 [5.6–6.6]	0.623
Fasting glucose, mg/dL [IQR]	96 [87–113]	107 [94–126]	0.003
Total cholesterol, mg/dL [IQR]	158 [133–188]	169 [145–199]	0.113
White blood cell, x 10^3^/uL [IQR]	7.40 [5.78–9.37]	8.64 [6.09–11.07]	0.013
High-sensitivity CRP, mg/dL [IQR]	0.20 [0.07–0.60]	0.26 [0.08–1.98]	0.199
Initial D-dimer, mg/L [IQR]	1.10 [0.74–1.80]	1.51 [1.02–3.36]	0.001
Follow-up D-dimer, mg/L [IQR]	1.11 [0.72–2.01]	2.14 [1.20–4.26]	< 0.001
Change in D-dimer, mg/L [IQR]	-0.03 [-0.31 -0.33]	0.09 [-0.78-1.71]	0.357

END = early neurological deterioration, NIHSS = National Institutes of Health Stroke Scale, CRP = c-reactive protein

**Table 4:** Multivariable logistic regression analysis to evaluate the effects of initial and follow-up D-dimer levels on early neurological deterioration

	Crude OR (95% CI)	*P*-value	Adjusted OR (95% CI)	*P*-value
**Initial D-dimer**				
Age	1.01 [0.98–1.04]	0.578	1.01 [0.98–1.04]	0.457
Male sex	0.65 [0.36–1.17]	0.150	0.78 [0.40–1.51]	0.459
Initial NIHSS score	1.06 [1.02–1.10]	0.002	1.03 [0.98–1.08]	0.203
Fasting glucose^*^	5.91 [1.97–17.78]	0.002	3.99 [1.15–13.89]	0.030
WBC counts^*^	2.96 [1.33–6.58]	0.008	2.20 [0.83–5.82]	0.114
Initial D-dimer^*^	1.61 [1.20–2.16]	0.001	1.48 [1.06–2.05]	0.020
**Follow-up D-dimer**				
Age	1.01 [0.98–1.04]	0.578	1.01 [0.98–1.04]	0.504
Male sex	0.65 [0.36–1.17]	0.150	0.79 [0.40–1.53]	0.483
Initial NIHSS score	1.06 [1.02–1.10]	0.002	1.03 [0.98–1.08]	0.226
Fasting glucose^*^	5.91 [1.97–17.78]	0.002	3.86 [1.09–13.64]	0.036
WBC counts^*^	2.96 [1.33–6.58]	0.008	2.13 [0.81–5.61]	0.127
Follow-up D-dimer^*^	1.79 [1.33–2.41]	< 0.001	1.60 [1.16–2.20]	0.004

NIHSS = National Institutes of Health Stroke Scale, WBC = white blood cell

^*^These variables were transformed into a log scale

**Fig. 1:**
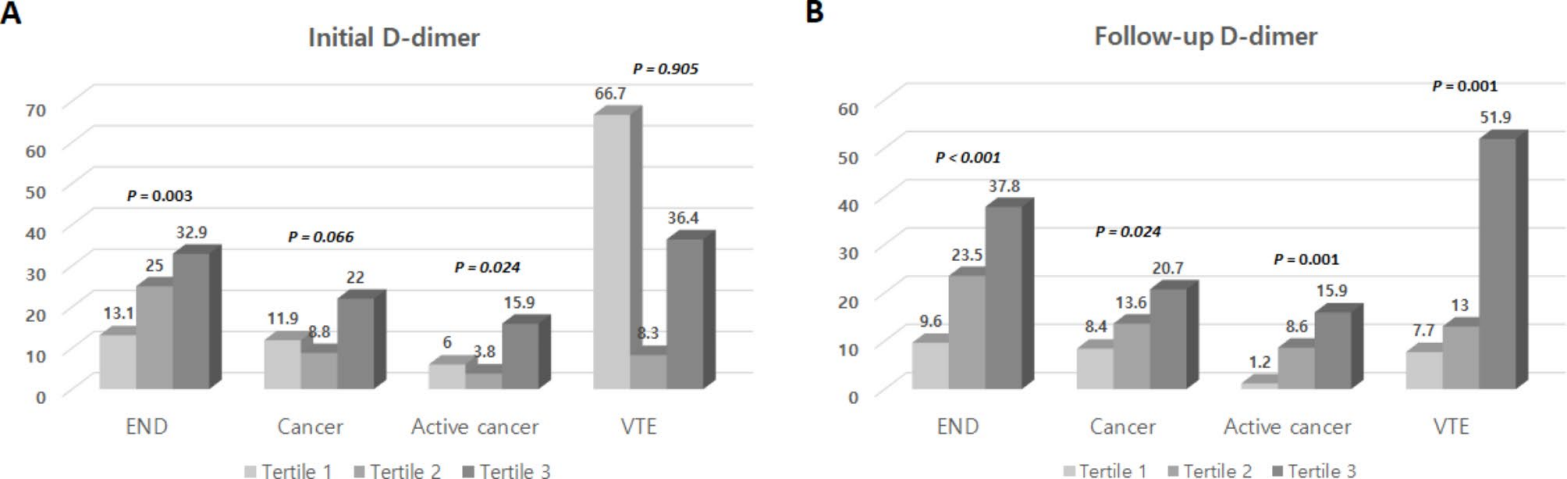
Quantitative relationship between initial/follow-up D-dimer level and early neurological deterioration, cancer, and venous thromboembolism using tertile values END = early neurological deterioration, VTE = venous thromboembolism. Initial D-dimer level showed a clear positive quantitative relationship with END (*P* = 0.003) and active cancer (*P* = 0.024). On the other hand, follow-up D-dimer showed a clear positive quantitative relationship with all of END (*P* < 0.001), history of cancer (*P* = 0.024), active cancer (*P* = 0.001), and VTE (*P* = 0.001).

Among our study population, a total of 63 patients were evaluated for clinically suspected VTE, and 18 (28.6%) of them were diagnosed. Regarding the analysis of the association between D-dimer levels and underlying cancer or VTE, the initial D-dimer level showed a statistically significant positive relationship only with active cancer (*P* = 0.024). On the other hand, the follow-up D-dimer level was found to be statistically significantly associated with a history of cancer (*P* = 0.024), active cancer (*P* = 0.001), and VTE (*P* = 0.001; Fig. 1).

## Discussion

In this study, we found that both the initial and follow-up D-dimer levels were associated with END in patients with AIS. In particular, the follow-up D-dimer level showed a close correlation not only with END, but also with underlying cancer or VTE. Therefore, if the initial D-dimer test is positive, measuring follow-up D-dimer levels within 7 days may help identify AIS patients who require close monitoring for END as well as those who require evaluation for underlying cancer or VTE.

The prevalence of END in our study population was 23.6%. Considering that the frequency of END among general stroke patients is 10–14%, this is quite a high number [30, 31]. This high prevalence may have occurred because many patients with small-vessel occlusion stroke with a relatively low initial D-dimer level were excluded. However, even considering these points, we believe that measuring the initial D-dimer level as a screening test to predict the high-risk group for END occurrence in AIS patients can be clinically helpful. In particular, as the enrollment standard for our study population was not as high as 0.55 mg/L, the slightly elevated initial D-dimer level should not also be overlooked.

The exact mechanism underlying the close association between initial D-dimer levels and END is unknown. However, several plausible hypotheses have been proposed. First, a high D-dimer level indicates overactivation of the coagulation and fibrinolytic system [3, 12, 17, 18]. In such an environment, additional thrombi form easily, enabling early stroke recurrence through various mechanisms such as embolism, in situ thrombosis, and instability of atherogenic plaque [3]. Second, high D-dimer levels may indicate large and severe strokes. D-dimer has been shown to be positively correlated with infarct volume and severity of neurological deficits [1, 14, 15, 17, 29]. Since initial stroke severity is the most potent predictor of END, a patient with a high D-dimer level may have severe stroke and experience frequent END. However, the initial stroke severity showed a biological interaction on END occurrence only with the initial D-dimer level, but not with the follow-up D-dimer level (Additional file 1: Table S1). Therefore, in patients with a characteristically high follow-up D-dimer level, the primary mechanism is thought to be additional thrombus formation by systemic hypercoagulability rather than this mechanism. Last, D-dimer itself can activate the inflammation cascade [12]. D-dimer can stimulate monocytes to secrete IL-6, which in turn causes local and systemic inflammation to aggravate neurological symptoms [3, 17].

Follow-up D-dimer levels also showed a clear correlation with END. However, we believe that the interpretation of this result should differ from that of the initial D-dimer level. Considering the temporal causal relationship, in many patients, END events occurred before the follow-up D-dimer level was measured. This is not surprising, as END events tend to occur more frequently immediately after a stroke. Therefore, unlike the initial D-dimer level, the follow-up D-dimer level seems inappropriate as a predictor of END. Rather than that, we found that the follow-up D-dimer level could be used as an indicator of stroke etiology or underlying pathological conditions that are likely to cause END. Indeed, follow-up D-dimer levels showed a close association with other determined stroke mechanisms, underlying cancer, and VTE in our data. In addition, it also showed reliable results in the analysis of the area under the curve of the receiver operating characteristic curve: 0.763 (0.666 to 0.861) and VTE: 0.803 (0.689 to 0.917). Underlying cancer and VTE are frequent in stroke patients and are difficult to detect because most of them are asymptomatic; but, they have a significant long-term impact on the patient’s prognosis [32–35]. Therefore, if continuous elevation is seen during follow-up in AIS patients for whom the initial D-dimer test was positive, broad evaluation to detect cancer or VTE may be necessary. In particular, our data showed no significant difference in the frequency of underlying cancer or VTE depending on the stroke mechanism, except for other determined strokes; therefore, our findings seem applicable to all AIS patients regardless of the mechanism.

There are several limitations when interpreting our findings. First, this was a retrospective cross-sectional study. Our findings suggest a close association between D-dimer level and END; however, this does not imply a causal relationship. Second, a selection bias should be considered. Follow-up D-dimer levels were measured only in patients whose initial D-dimer exceeded 0.55 mg/L at our center, and were included in this study. Therefore, the initial D-dimer level in our study population will be higher than that in general AIS patients, which may lead to an underestimation of the influence of the initial D-dimer level. Third, the results related to VTE should be interpreted with caution. Because the attending physician conducted VTE-related evaluations when the patient was clinically suspected, relevant data exists only for some patients. This can be considered as a limitation of the retrospective study design. Fourth, it should be noted that other medical conditions that may contribute to the elevation of D-dimer should also be considered (e.g., sepsis, chronic inflammation, heart failure) [28]. Last, we used a relatively sensitive definition of END [36]. However, this definition has often been used in previous studies as well. Moreover, the END group showed a higher discharge modified Rankin Scale score than the non-END group (4 [3–5] versus 2 [0–2], *P* < 0.001). Therefore, the END that we used could be interpreted as clinically meaningful.

## Conclusion

In conclusion, we demonstrated that both the initial and follow-up D-dimer levels were closely associated with the clinical course of AIS. D-dimer is a parameter that is widely used for AIS patients in the clinical field, because it is resistant to the ex vivo environment, relatively stable, and inexpensive [29]. Therefore, by measuring the initial D-dimer level as a screening test in AIS patients and measuring the follow-up D-dimer level in high-risk patients who have high levels, it may be possible to predict the risk of early prognosis and complications. Of course, our findings should be validated by future prospective studies.

## Electronic supplementary material

Below is the link to the electronic supplementary material.

**Additional file 1: Table S1.** Items of National Institutes of Health Stroke Scale.
**Additional file 2: Table S2.** Trial of Org 10,172 in Acute Stroke Treatment (TOAST) classification.
**Additional file 3: Table S3.** Biological interaction between initial/follow-up D-dimer levels and initial NIHSS score for early neurological deterioration.

## Data Availability

All data related to this study are included in the main text and the additional files.

## Abbreviations

ESUS: Embolic stroke of undetermined source
END: Early neurological deterioration
AIS: Acute ischemic stroke
NIHSS: National Institutes of Health Stroke Scale
VTE: Venous thromboembolism
WBC: White blood cell
hs-CRP: High-sensitivity C-reactive protein
ROC: Receiver operating characteristic
