# Supplementary material for: Clinical significance of D-dimer levels during acute period in ischemic stroke
Source: Thromb J. 2023 May 9;21:55. doi: 10.1186/s12959-023-00496-1 (PMC10170762; doi:10.1186/s12959-023-00496-1)
Supplement: Supplementary file 1 — Additional file 1: Table S1. Items of National Institutes of Health Stroke Scale. Additional file 2: Table S2. Trial of Org 10,172 in Acute Stroke Treatment (TOAST) classification. Additional file 3: Table S3. Biological interaction between initial/follow-up D-dimer levels and initial NIHSS score for early neurological deterioration. [file 12959_2023_496_MOESM1_ESM.docx]

**Additional file 1. Items of National Institutes of Health Stroke Scale**

- 1a. Level of consciousness
  - Alert, keenly responsive (0)
  - Not alert, but arousable by minor stimulation (1)
  - Not alert, requires repeated stimulation (2)
  - Unresponsive or responds only with reflex (3)
- 1b. Level of consciousness questions
  - What is the months? (1)
  - What is your age? (1)
- 1c. Level of consciousness commands
  - Open and close your eyes (1)
  - Grip and release your hand (1)
- 2. Best gaze
  - Normal (0) Partial gaze palsy (1) Forced deviation (2)
- 3. Visual
  - No visual loss (0) partial hemianopsia (1) complete hemianopsia (2) bilateral hemianopsia (2)
- 4. Facial palsy
  - Normal (0) minor paralysis (1) partial paralysis (2) complete paralysis of 1 or both sides (3)
- 5. Motor-arm (left and right, respectively)
  - No drift (0)
  - Drift (1)
  - Some effort against gravity (2)
  - No effort against gravity; limb falls (3)
  - No movement (4)
- 6. Motor-leg (left and right, respectively)
  - No drift (0)
  - Drift (1)
  - Some effort against gravity (2)
  - No effort against gravity (3)
  - No movement (4)
- 7. Limb ataxia
  - Absent (0) Present in 1 limb (1) Present in 2 limbs (2)
- 8. Sensory
  - Normal; no sensory loss (0)
  - Mild-to-moderate sensory loss (1)
  - Severe to total sensory loss (2)
- 9. Best language
  - No aphasia; normal (0)
  - Mild to moderate aphasia (1)
  - Severe aphasia (2)
  - Mute, global aphasia (3)
- 10. Dysarthria
  - Normal (0) mild to moderate dysarthria (1) severe dysarthria (2)
- 11. Extinction and inattention
  - No abnormality (0)
  - Visual, tactile, auditory, spatial, or personal inattention (1)
  - Profound hemi-inattention or extinction (2)

**Total score = 0-42**

**Additional file 2. Trial of Org 10172 in Acute Stroke Treatment (TOAST) classification**

- Large-artery atherosclerosis (embolus/thrombosis)
  - Significant (> 50%) stenosis or occlusion of a major brain artery or branch cortical artery
- Small-vessel occlusion (lacunae)
  - Relevant brainstem or subcortical hemispheric lesion with a diameter of less than 1.5 cm
- Cardio-embolism
  - **Medium risk:** mitral valve prolapse, mitral annulus calcification, mitral stenosis without atrial fibrillation, left atrial turbulence, atrial septal aneurysm, patent foramen ovale, atrial flutter, lone atrial fibrillation, bioprosthetic cardiac valve, nonbacterial thrombotic endocarditis, congestive heart failure, hypokinetic left ventricular segment, myocardial infarction (4 weeks ~ 6 months)
  - **High risk:** mechanical prosthetic valve, mitral stenosis with atrial fibrillation, atrial fibrillation, left atrial/atrial appendage thrombus, sick sinus syndrome, recent myocardial infarction (< 4 weeks), left ventricular thrombus, dilated cardiomyopathy, akinetic left ventricular segment, atrial myxoma, infective endocarditis
- Stroke of other determined etiology
  - Nonatherosclerotic vasculopathy, hypercoagulable state, hematologic disorder, active cancer
- Stroke of undetermined etiology
  - Two or more causes identified
  - Negative evaluation (cryptogenic)
  - Incomplete evaluation

**Additional file 3. Biological interaction between initial/follow-up D-dimer levels and initial NIHSS score for early neurological deterioration**

|  | **Initial D-dimer** | | **Follow-up D-dimer** | |
| --- | --- | --- | --- | --- |
|  | Adjusted OR  (95% CI) | *P*-value | Adjusted OR  (95% CI) | *P*-value |
| Age | 1.01 [0.98-1.04] | 0.484 | 1.01 [0.98-1.04] | 0.529 |
| Male sex | 0.79 [0.41-1.54] | 0.488 | 0.78 [0.40-1.53] | 0.474 |
| Initial NIHSS score | 1.06 [1.01-1.12] | 0.028 | 1.05 [0.99-1.12] | 0.094 |
| Fasting glucose^*^ | 4.71 [1.33-16.71] | 0.016 | 4.34 [1.21-15.56] | 0.024 |
| WBC counts^*^ | 2.19 [0.82-5.84] | 0.118 | 2.12 [0.80-5.60] | 0.131 |
| Initial D-dimer^*^ | 2.09 [1.30-3.34] | 0.002 | … | … |
| Follow-up D-dimer^*^ | … | … | 1.89 [1.22-2.93] | 0.004 |
| Interaction [initial NIHSS x initial D-dimer] | 0.96 [0.93-1.00] | 0.047 | … | … |
| Interaction [initial NIHSS x follow-up D-dimer] | … | … | 0.98 [0.93-1.02] | 0.256 |

NIHSS = National Institutes of Health Stroke Scale, WBC = white blood cell

^*^These variables were transformed into a log scale
